# Transcriptomic Analysis of the Spleen from Asian Seabass (*Lates calcarifer*) Infected with Infectious Spleen and Kidney Necrosis Virus

**DOI:** 10.3390/v17050728

**Published:** 2025-05-19

**Authors:** Hong-Yi Xin, Lim Xin Ying, Lee Ching Pei Carmen, Mookkan Prabakaran

**Affiliations:** Temasek Life Sciences Laboratory, 1 Research Link, National University of Singapore, Singapore 117604, Singapore; hongyi@tll.org.sg (H.-Y.X.); xinying@tll.org.sg (L.X.Y.); carmen@tll.org.sg (L.C.P.C.)

**Keywords:** infectious spleen and kidney necrosis virus, transcriptome sequencing, differentially expressed genes, key genes, hub genes, Asian seabass

## Abstract

Infectious spleen and kidney necrosis virus (ISKNV) is an emerging viral pathogen with an expanding host range, posing a significant threat to economically important fish species. In this study, we isolated the ISKNV strain responsible for disease outbreaks in Asian seabass (*Lates calcarifer*) and analyzed the transcriptomic profile of spleen tissues from experimentally infected fish. The phylogenetic analysis confirmed that the virus belongs to clade I of ISKNV. Next-generation sequencing identified differentially expressed genes, providing a comprehensive overview of the transcriptional landscape in the spleen of ISKNV-infected fish. The pathway analysis revealed complex host–virus interactions, impacting immune regulation, endocytosis, cell communication, cell cycle arrest, and programmed cell death. To further investigate these interactions, we analyzed relevant pathways in the Reactome database for Asian seabass, humans, and zebrafish, constructed a protein–protein interaction (PPI) network using STRING database, and identified hub genes using six different algorithms. This analysis revealed 69 key genes, including 41 hub genes and 28 key genes that connect different pathways or clusters within the PPI network. These findings provide new insights into the molecular mechanisms driving ISKNV infection in Asian seabass. Future research should focus on elucidating the regulatory functions of these key genes and their roles in ISKNV pathogenesis.

## 1. Introduction

Infectious spleen and kidney necrosis virus (ISKNV), a member of the *Iridoviridae* family within the *Megalocytivirus* genus, is a large double-stranded DNA virus with an icosahedral symmetry. Its genome spans 111.4 kb and encodes 132 open reading frames (ORFs) [1]. ISKNV infects a wide range of hosts, primarily targeting the spleen, kidney, and other immune organs, leading to inflammation, necrosis and immunosuppression. Infected fish exhibit symptoms such as severe anemia, petechiae in the gills, and swelling of the spleen and kidney, along with histopathological features, including systemic cell enlargement and necrosis of splenocytes and hematopoietic cells [2]. The mandarin fish (*Siniperca chuatis*) is a primary host for ISKNV, though infections have also been observed in over 60 other species of both marine and freshwater fish, including economically important fish species in Asia, such as the Asian seabass (*Lates calcarifer*). ISKNV is one of the most significant causative agents of fish disease and is listed by the World Organization for Animal Health (OIE) as a notifiable pathogen.

Asian seabass (ASB) or barramundi, is an economically important catadromous fish species in aquaculture, belonging to the order Perciformes, with a broad distribution across the Asia-pacific region. The Asian seabass market is forecasted to grow at a compound annual growth rate of 4.5% from 2024 to 2034, with its market value expected to increase from USD 1012 million in 2024 to USD 1567 million by 2034 [3]. Despite its economic importance, there are no effective treatment measures to control ISKNV disease. Therefore, gaining a deeper understanding of ISKNV pathogenesis is crucial for developing new preventive and host-targeted therapeutic strategies.

The molecular mechanisms behind ISKNV pathogenesis and its interactions with hosts are still largely unknown, particularly in Asian seabass. Understanding viral–host interactions is critical, as many viruses encode proteins that mimic or modify host proteins to divert cellular resources toward viral replication.

In this study, we isolated the ISKNV strain responsible for mortality in Asian seabass and analyzed the transcriptomic profile of spleen tissues from experimentally infected fish. Based on the differentially expressed genes (DEGs) identified, key genes were further pinpointed from pathogenesis-related host cell pathways, including adaptive and innate immunity, cytokine signaling, interferon (IFN) signaling, Toll-like receptor (TLR) signaling, programmed cell death, cell communication, cell cycle arrest, and PI3K signaling pathways.

## 2. Materials and Methods

### 2.1. Virus Detection and Isolation

The liver, spleen, and kidney were collected from diseased Asian seabass (average bodyweight of 200 g) obtained from an aquaculture farm in Singapore in 2022. The collected tissue samples were immediately homogenized for DNA extraction using a Purelink Viral RNA/DNA Mini Kit (Invitrogen, Carlsbad, CA, USA). PCR analysis was performed using Megalocytivirus and ISKNV major capsid protein (MCP)-specific primers (Table S1) to confirm viral presence. The MCP gene was amplified, cloned into the pJet 1.2 using the cloneJET PCR cloning kit (Thermo Fisher Scientific, Waltham, MA, USA), and sequenced. Phylogenetic analysis was performed using the maximum likelihood method to compare the relationships among seven ISKNV strains from different clades. For virus isolation, spleen or kidney lysates were treated with 2× antibiotic–antimycotic (Gibco, Waltham, MA, USA) and centrifuged at 4000× *g* for 10 min to obtain the supernatant. The supernatant was filtered through a 0.45 µm membrane filter, freeze-thawed twice to release viral particles, and then incubated at 28° C with SAF-1 cell monolayers. SAF-1 cells, derived from the caudal fin tissue of gilt-head seabream (*Sparus aurata*, ECACC 00122301). The cells were monitored for cytopathic effects (CPEs) under a microscope. Infected cells were harvested on day 5 post-infection and stored at –80 °C. The virus was further plaque-purified to obtain a pure virus population as described below. A selected viral plaque was passaged on SAF-1 cells, and viral titer was determined by 50% tissue culture infectious dose (TCID_50_) assay in SAF-1 cells [4]. Briefly, 10-fold serially diluted viral culture samples were added onto the sub-confluent SAF-1 monolayers in a 96-well culture plate and incubated at 28 °C for 5 days. CPE was recorded, and viral titers were calculated according to the Reed and Muench method [5] and expressed as TCID_50_/mL.

### 2.2. Viral Plaque Purification

Briefly, the viral supernatant was subjected to a 1:10 serial dilution prior to infection of confluent SAF-1 cells in a 6-well plate for 2 h at 28 °C. The plates were gently rocked every 15 min during the incubation time. Following incubation, the inoculum was removed, and 4% low-melting agarose (Gibco, Waltham, MA, USA) with 1.5x-concentrated Leibovitz L-15 medium (Gibco, Waltham, MA, USA), supplemented with 15% fetal bovine serum (FBS, Gibco, Waltham, MA, USA), 2 mM L-Glutamine (Gibco, São Paulo, Brazil), and 1× antibiotic–antimycotic (Gibco, Waltham, MA, USA), was added to each well. After 4 days of incubation, the viral plaques were clonally isolated and cultured in SAF-1 cells.

### 2.3. Experimental Infection

Healthy Asian seabass (50 ± 5 g) were obtained from a commercial hatchery (Allegro Aqua, Singapore). During acclimatization, 10 fish were randomly selected and tested for ISKNV and other bacterial diseases common to the species. A total of 60 fish were evenly distributed into two 200 L recirculating tanks, each outfitted with standard biofiltration systems and aeration at Temasek Life Sciences Laboratory (TLL, Singapore). The fish were kept in UV-irradiated seawater at 30 ± 1 °C, with a 12:12 light and dark photoperiod cycle. All fish were fed with a commercial pellet diet at 5% of their body weight daily. Thirty fish (*n* = 30) were intraperitoneally injected with 10^5.5^ TCID_50_/mL, while the control group was injected with cell culture medium. The fish (*n* = 10) from each group were euthanized on day 7 post-infection. The spleen tissues were collected under RNase-free conditions and stored at −80 °C for transcriptomic analysis and qRT-PCR analysis.

### 2.4. RNA Isolation and Transcriptome Sequencing

Total RNA was extracted from both control and ISKNV-infected spleen tissues using the mirVana™ miRNA Isolation Kit (Thermo Fisher Scientific, Vilnius, Lithuania), according to the manufacturer’s protocol. The RNA concentration was measured using a nanodrop2000 (Thermo Fisher Scientific, Waltham, MA, USA); the RNA integrity was analyzed with an Agilent 2100 Bioanalyzer (Agilent Technologies, Inc., Santa Clara, CA, USA), ensuring that all samples met the quality control criteria (RIN ≥ 8) before proceeding with Illumina library preparation. The cDNA libraries were created using the TruSeq Stranded mRNA Library Prep Kit (Illumina, San Diego, CA, USA). Paired-end 150 bp (PE150) sequencing was carried out on an Illumina NovaSeq 6000 sequencer. Library preparation and sequencing were conducted by Macrogen, Inc. (Seoul, Republic of Korea).

### 2.5. Analysis of DEGs

For sequencing results, Trimmomatic was used to remove adapter sequences and trim bases with a quality score lower than 3 from the ends of reads. The sliding window method was applied with a window size of 4 and a minimum mean quality score of 15. Bases within a window that did not meet this quality threshold were trimmed. After trimming, reads shorter than 36 base pairs (bp) were discarded. HISAT2 was used to map the reads to the TLL_Latcal_v3 genome (GCF_001640805.2) with the proprietary parameters of Macrogen Pte. Ltd., Singapore. Library normalization was conducted using trimmed mean of M-values (TMM) method. To reduce systematic bias, size factors were estimated from the read count data via calcNormFactors method, and the read count data was subsequently normalized using the TMM method in edgeR library. Normalization values, calculated based on transcript length and depth of coverage, are provided as FPKM (Fragments Per Kilobase of transcript per Million Mapped reads)/RPKM (Reads Per Kilobase of transcript per Million mapped reads) and TPM (Transcripts Per Kilobase Million). Differentially expressed genes (DEGs) in ISKNV-infected spleen samples were identified by comparison with healthy controls. DEGs were determined using edgeR, and the statistical analysis was performed with the exactTest function in EdgeR, which conducts pair-wise tests for differential expression between two groups on negative binomially distributed counts.

The differential expression analysis was performed based on the fold change (FC) of normalized read counts between the infected and control groups. Transcripts with an absolute FC greater than 2 and a false discovery rate (FDR) ≤ 0.05 were considered significantly differentially expressed. The DEG lists were functionally annotated using the DAVID bioinformatics tool, which inherently maps gene identifiers to associated protein products when performing enrichment analysis based on databases. DAVID’s internal algorithms and statistical tests were used to define the significance of the functional annotation. The identified DEGs were further classified into biological pathways using the manually curated Reactome server (https://reactome.org/ (accessed on 14 December 2024) [6]. For gene segregation in REACTOME, a homology analysis between Asian seabass and Zebrafish was first performed in ENSEMBL and orthoDB (Table S2a). The gene symbol list (Table S2b) of all genes homologous to those of zebrafish was input into the REACTOME server for pathway analysis. The genes were separately projected to all the species and human (all non-human identifiers are converted to their human equivalents). Based on the results from zebrafish and human species, the genes were grouped into pathways related to the innate immune response, adaptive immune response, antigen processing and presentation, cytokine-mediated immune response, IFN signaling, TLR signaling, cell death, cell communication, cell cycle arrest, endocytosis, and the PI3K pathway.

### 2.6. Identification and Visualization of Hub Genes

To establish the protein–protein interaction (PPI) network, the online tool STRING (https://string-db.org/ (accessed on 7 January 2025) was used with a high confidence interaction score of 0.7 and a medium FDR stringency of 5%. The Cytoscape software (version 3.10.3) was employed to visualize the PPI network. Hub genes within the PPI network were identified using the cytoHubba plugin in Cytoscape, applying six different calculation methods, which include maximal clique centrality (MCC), degree, maximum neighborhood component (MNC), node connect closeness, betweenness, and stress. The common genes identified among the top 10 ranked genes across these methods were classified as hub genes.

### 2.7. Validation of the DEGs by RT-qPCR

To validate the transcriptomic data, immune-related DEGs were selected for quantitative real-time PCR (qRT-PCR) analysis. Specific primers were designed using Primer-BLAST (https://www.ncbi.nlm.nih.gov/tools/primer-blast/), with 18S rRNA served as an internal control (Table 1). The qRT-PCR reactions were performed in a 10 μL reaction volume, consisting of 0.5 μL cDNA, 0.5 μL each of forward and reverse primers, 5 μL GoTaq^®^ qPCR Master Mix (2X), and 3 μL nuclease-free water. The cycling program included an initial denaturation at 95 °C for 3 min, followed by 40 cycles of denaturation at 95 °C for 10 s, annealing at 60 °C for 15 s, and extension at 60 °C for 20 s. No-template controls were included to rule out contamination. All reactions were carried out in triplicate for two biological replicates to ensure reproducibility.

### 2.8. Statistical Analysis

Statistical analyses were performed using GraphPad Prism (version 8.0.2). Inter-group comparisons were calculated using the Wilcoxon tests. Statistical significance was set at two-sided *p* < 0.05.

## 3. Results

### 3.1. Isolation and Verification of ISKNV

**Figure 1:**
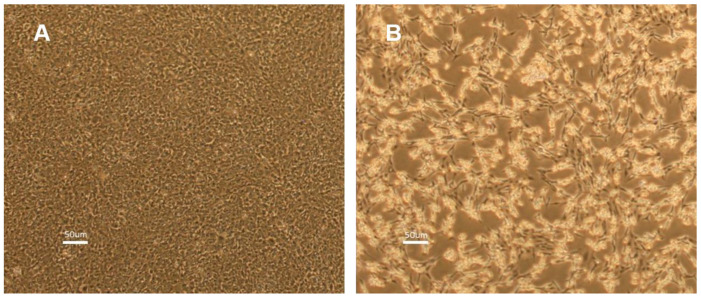
SAF-1 cells infected with ISKNV. (**A**). Uninfected SAF-1 cells (control) on day 2. (**B**) ISKNV-infected SAF-1 cells showing cytopathic effects on day 2 post-infection at 28 °C.

**Figure 2:**
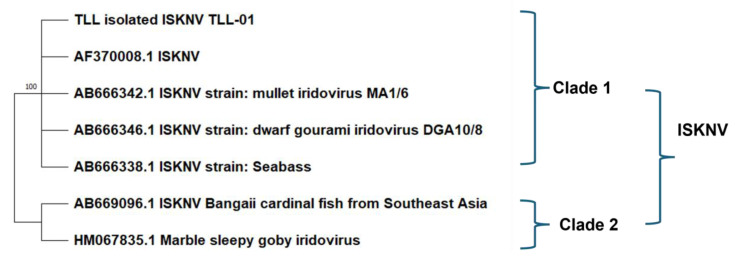
Phylogenetic tree for representative ISKNV genotype clade and the Asian seabass ISKNV isolate (TLL-01, GenBank accession number PV101205), calculated by the maximum likelihood method.

ISKNV was successfully isolated from the kidney of diseased ASB and propagated in SAF-1 cells. The CPEs characterized by cell rounding were observed within two days post-infection (Figure 1). The virus was plaque-purified to obtain pure virus population. After five serial passages, the virus reached a titer >10^6.5^ TCID_50_/_mL_ on day 5. The MCP gene was confirmed by sequencing, and the phylogenetic analysis revealed that ISKNV strain (GenBank accession number PV101205) belongs to genotype Clade 1 (Figure 2).

### 3.2. DEG Analysis in Spleens of ISKNV-Infected and Healthy Asian Seabass

**Figure 3:**
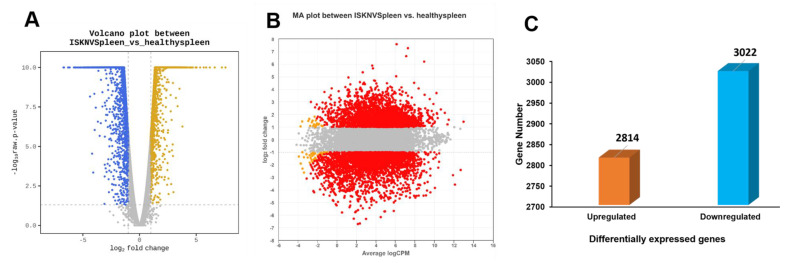
Visualization of DEGs between ISKNV-infected and healthy ASB spleen tissues. (**A**) MA plot of the DEGs, where each point represents a gene. The *X*-axis represents log_2_ (counts per million), while the *Y*-axis represents log_2_ (fold change). (**B**) Volcano plot showing DEGs between ISKNV-infected and healthy ASB spleens. The *X*-axis represents log_2_ (fold change), and the *Y*-axis represents -log_10_(FDR). The *Y*-axis corresponds to the -log_10_ of adjusted *p*-values for all read counts in each comparison. (**C**) Bar chart of upregulated and downregulated DEGs in ISKNV-infected and healthy ASB spleens.

**Figure 4:**
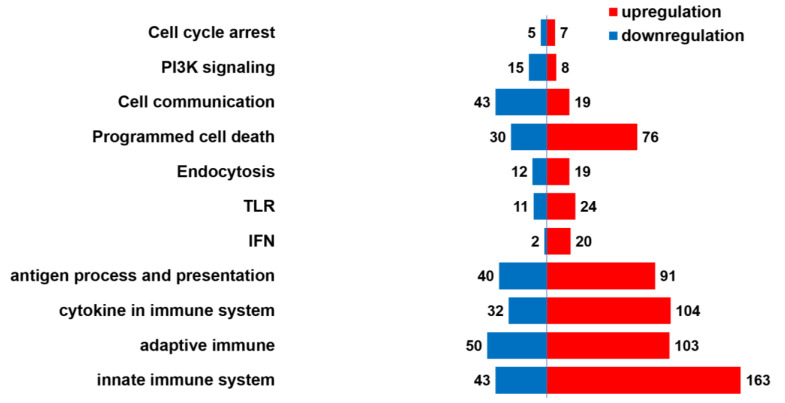
Segregation of the DEGs according to signaling pathways in the reactome database. DEGs were categorized based on their association with signaling pathways related to pathogen infection.

DEG analysis was performed to compare the expression profiles of spleen tissues from ISKNV-infected and healthy Asian seabass. A total of 5836 DEGs were identified (*p* < 0.05). Among these, 2814 genes were upregulated (positive log_2_FC), while 3022 genes were downregulated in infected samples (Figure 3 and Table S3). The segmentation of the DEGs by REACTOME hit the pathways related to virus pathogenesis (Figure 4, Table S4).

### 3.3. DEGs Involved in Immune Response

**Figure 5:**
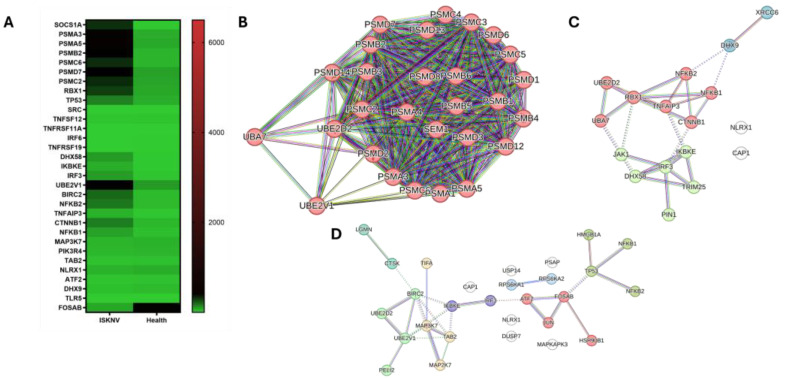
Key genes in immune response. (**A**) Heatmaps in TPM illustrating the expression levels of key immune-related genes. (**B**) PPI network of shared genes involved in antigen processing and presentation, adaptive immunity, innate immunity, and cytokine pathways. (**C**) PPI network of the IFN pathway. Red nodes represent the cluster of interferon-alpha/beta induction; green nodes represent the cluster of RIG-I-like receptor signaling pathway. (**D**) PPI network of TLR pathway. Red nodes represent the cluster of transcription factors; green nodes represent the cluster of JNK phosphorylation and activation; olive nodes represent the cluster of NF-kB activation; purple nodes represent the cluster of cytosolic DNA-sensing pathway; light sky-blue nodes represent the cluster of RSK activation.

In our transcriptional analysis, 369 DEGs were identified as relevant to immunological processes, including innate and adaptive immunity, antigen processing and presentation, cytokine signaling, the Toll-like receptor (TLR) pathway, and the interferon (IFN) pathway. Among these, 136 genes were associated with cytokine responses, 131 with antigen processing and presentation, 153 with the adaptive immune system, and 206 with the innate immune system. A total of 31 genes were common to the adaptive immune system, innate immune system, antigen processing and presentation, and cytokine signaling pathways. The expression patterns of selected DEGs, measured via NGS transcriptomic data, are depicted in the heatmap (Figure 5A). Furthermore, key hub genes in the protein–protein interaction (PPI) network (Figure 5B, Table S5) were identified as PSMA3, PSMA5, PSMB2, PSMC2, PSMC6, and PSMD7. Interestingly, the PPI analysis in the cytokine signaling in immune response revealed interactions between TNFRSF11A and TNFSF12 in humans, with TNFSF12 also interacting with BIRC2. In Asian seabass, TNFSF12 interacts with BIRC2, a hub gene in the TLR pathway. SOCS1A was clustered within the interferon signaling cluster and connected to the proteasome cluster via the RBX1 gene, the apoptosis cluster through TP53, and the VEGF pathway through SRC. In addition, the disconnected genes, TNFRSF19 and IRF6, were downregulated and may influence ISKNV infection in Asian seabass. Hub genes in the interferon pathway included RBX1, TNFAIP3, DHX58, NFKB2, IKBKE, NFKB1, IRF3, and CTNNB1 (Figure 5C, Tables S6 and S7). In the TLR pathway, the hub genes ATF2, BIRC2, FOSAB, IKBKE, MAP3K7, TAB2, and UBE2V1 were identified (Figure 5D, Tables S8 and S9). The two genes PIK3R4 and TLR5 were not observed in the PPI network of Asian seabass, zebrafish, and humans. In our study, we observed that the gene PIK3R4 was upregulated, whereas TLR5 was downregulated.

### 3.4. DEGs Involved in Endocytosis

**Figure 6:**
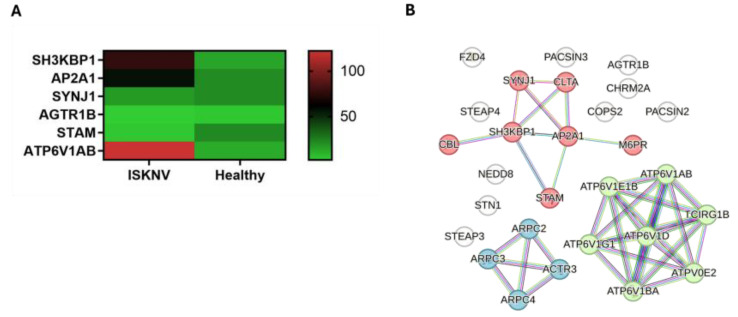
Key genes in the endocytosis pathway. (**A**) Heatmaps in TPM illustrating the expression levels of key genes in the endocytosis pathway. (**B**) PPI network of the DEGs in the endocytosis pathway. Red nodes represent the cluster of negative regulation of MET activity; green nodes represent the cluster of ROS and RNS production in phagocytes; blue nodes represent the cluster of actin nucleation.

Among the DEGs, 31 genes are associated with endocytosis, all of which are part of clathrin-mediated endocytosis. Among them, 14 genes are specifically involved in cargo recognition. The expression of the key DEGs is presented in the heatmap (Figure 6A). The hub genes in the PPI network (Figure 6B, Tables S10 and S11) were identified as AP2A1 and SH3KBP1. The network clustering identified three functional groups, The first group, the negative regulation of MET activity, includes the genes AP2A1, SH3KBP1, SYNJ1, and STAM. The second group, ROS and RNS production in phagocytes, comprises members of the ATP6V1 family, such as ATP6V1AB. Lastly, the third group, Arp2/3 complex-mediated actin nucleation, includes the gene AGTR1b. These clusters might have the potential roles of identified DEGs in ISKNV pathogenesis.

### 3.5. DEGs Involved in Cell–Cell Communication

**Figure 7:**
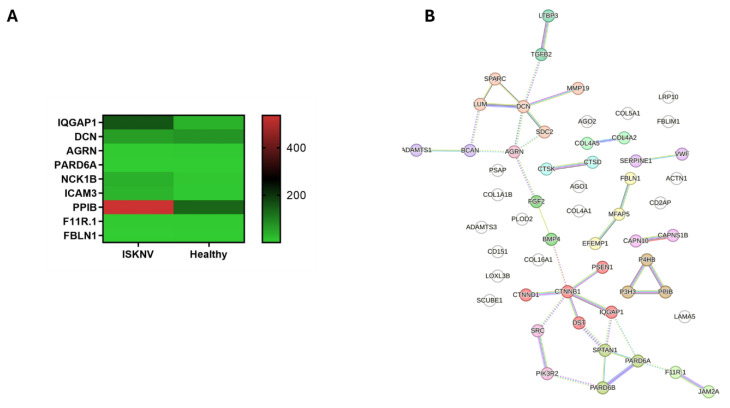
Key genes in the cell–cell communication pathway. (**A**) Heatmaps in TPM illustrating the expression levels of key genes involved in the cell–cell communication pathway. (**B**) PPI network of the DEGs in cell–cell communication pathway. Red nodes represent the cluster of RHO GTPases that activate IQGAPs; brown nodes represent the cluster of collagen binding; dark golden rod nodes represent the cluster of collagen biosynthesis; olive nodes represent the cluster of PB1 domain; green nodes represent the cluster of leukocyte cell–cell adhesion.

Cell–cell communication is essential in viral infections, as viruses exploit these pathways to facilitate entry, replication, and dissemination while evading host defenses. In this study, a total of 62 DEGs were associated with the cell–cell communication pathway, with five genes including AGO2, CTNNB1, AGO1, CDH19, and CTNND1 functioning in both cell junction and adhesion processes. PPI analysis classified these genes into six main clusters: IQGAP activation, collagen binding, collagen biosynthesis, molecules associated with elastic fibers, PB1 domain, and leukocyte cell–cell adhesion. The hub genes identified include PARD6A, DCN, AGRN, and IQGAP1, distributed across the IQGAP activation, collagen binding, and PB1 domain clusters (Figure 7B, Tables S12 and S13). Additionally, key genes—PPIB (also known as cyclophilin B) in the collagen biosynthesis cluster, FBLN1 (also as fibulin-1) in the cluster molecules associated with elastic fibers and, F11r.1 (also as JAMA) in the leukocyte cell–cell adhesion cluster—were selected based on their known function in virus infection. Seven genes that were present in zebrafish were absent in Asian seabass, with only NCK1B and ICAM3 being upregulated in the NGS results. Studies have shown that ICAM3 may negatively regulate T cell activities involved in *viral* replication [7]. The expression levels of these key genes are presented in the heatmap (Figure 7A).

### 3.6. DEGs Involved in the Programmed Cell Death

**Figure 8:**
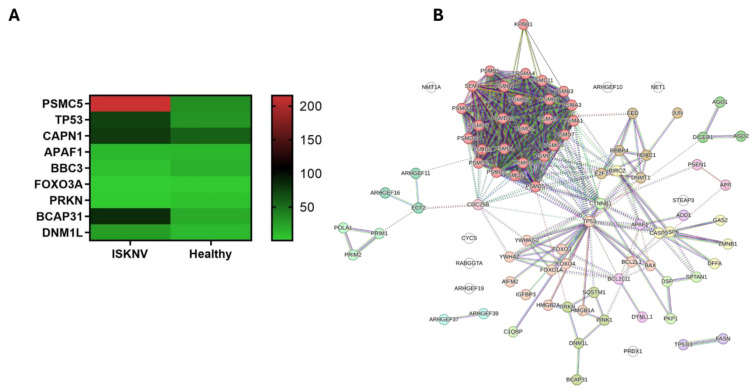
Key genes in the programmed cell death pathway. (**A**) Heatmaps in TPM illustrating the expression levels of key genes in programmed cell death pathway. (**B**) PPI network of the DEGs in programmed cell death pathway. Red nodes represent the cluster of proteasome; brown nodes represent the cluster of p53 signaling; yellow nodes represent the cluster of apoptotic execution phase; olive nodes represent the cluster of mitophagy; dark golden nodes represent the cluster of ESC/E(Z) complex.

Programmed cell death (PCD) plays a critical role in maintaining cellular homeostasis and defending against pathogens, with viruses often exploiting PCD pathways to enhance their replication and lifecycle. In this study, a total of 106 DEGs were associated with programmed cell death in ISKNV-infected spleen, including 79 genes related to apoptosis and 4 genes to necroptotic cell death. Among these, PRKN was the only downregulated necroptosis-related gene. In our study, the 11 proapoptotic genes were differentially expressed, in which four DEGs, APAF1, BBC3, FOXO3A, and CAPN1 were downregulated in ISKNV-infected spleen. Interestingly, the BCL2L1 gene produces two distinct isoforms. The longer isoform, Bcl-xL, functions as an anti-apoptotic protein, while the shorter isoform, Bcl-xS, exhibits pro-apoptotic properties. In our study, we observed a downregulation of BCL2L1. It is essential to determine which isoform is predominant. The expression levels of the key genes in PCD pathway are presented in the heatmap (Figure 8A). The PPI network identified five distinct clusters: proteasome, p53 signaling pathway, ESC/E(Z) complex, apoptotic execution phase, and mitophagy (Figure 8B, Tables S14 and S15). Hub genes included TP53 in the p53 signaling pathway cluster and PSMC5 in the proteasome clusters. In the mitophagy cluster, DNM1L and BCAP31 were upregulated. Studies have shown that some viruses activate the mitophagy pathway to promote their own replication [8].

### 3.7. DEGs Involved in Cell Cycle Arrest

**Figure 9:**
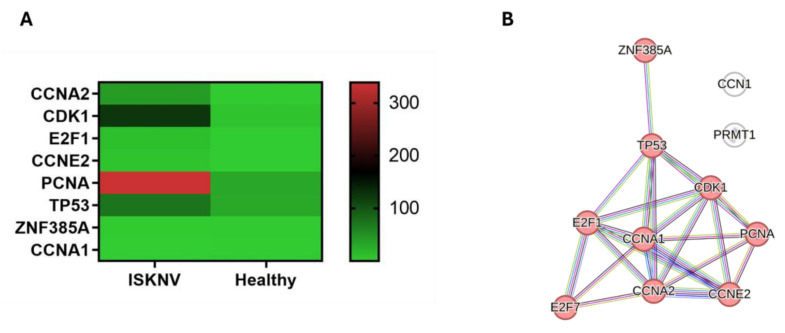
Key genes in the cell cycle arrest pathway. (**A**) Heatmaps in TPM illustrating the expression levels of key genes associated with the cell cycle arrest pathway. (**B**) PPI network of the DEGs in the cell cycle arrest pathway. Red nodes represent cluster of TP53-regulated transcription of cell cycle genes.

In our DEGs, 12 genes are involved in cell cycle arrest. The genes involved in G1 cell cycle arrest included CCN1, CCNA1, CCNA2, CCNE2, CDK1, E2F1, and E2F7, while PRMT1, CDK1, PCNA, and GADD45AB were involved in G2 cell cycle arrest. The genes TP53 and ZNF385A were involved in both G1 and G2 cell cycle arrest. In addition, TP53 and E2F1 were also involved in cell apoptosis. The expression level of the cell cycle arrest genes is presented in the heatmap (Figure 9A). PPI analysis identified the hub genes CCNE2, E2F1, CCNA2, TP53, CCNA1, PCNA, and CDK1. All are associated with the transcription regulation of cell cycle genes (Figure 9B, Tables S16 and S17).

### 3.8. DEGs Involved in PI3K/AKT Signaling Pathways

**Figure 10:**
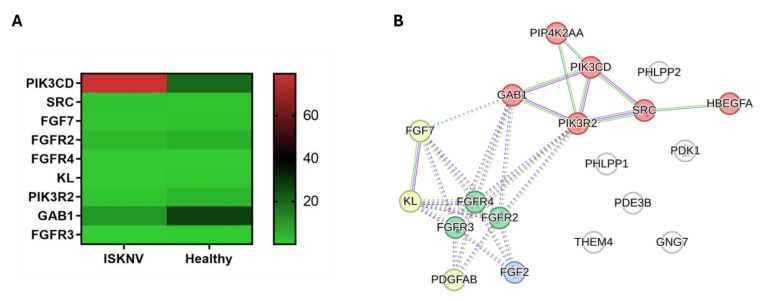
Key genes in the PI3K pathway. (**A**) Heatmaps in TPM illustrating the expression levels of key genes associated with the PI3K pathway. (**B**) PPI network of the DEGs in the PI3K pathway. Red nodes represent the cluster of ErbB signaling; green nodes represent the cluster of regulation of hematopoietic stem cell differentiation.

The phosphatidylinositide 3-kinase (PI3K) signaling pathway is integral to various cellular processes, including growth, proliferation, metabolism, and migration. During virus infections, this pathway can influence viral entry, replication, and immune evasion. Among the DEGs, 23 genes were found to be associated with the PI3K signaling pathway. The expression levels of the key genes involved in this pathway are illustrated in the heatmap (Figure 10A). The PPI analysis identified FGF7, FGFR2, FGFR3, FGFR4, GAB1, KL, PIK3CD, PIK3R2, and SRC as hub genes. Of these, GAB1, PIK3CD, PIK3R2, and SRC were grouped within the ErbB signaling pathway group, while FGFR2, FGFR3, and FGFR4 were categorized under the regulation of hematopoietic stem cell differentiation (Figure 10B, Tables S18 and S19).

### 3.9. Validation of the Selected DEGs

**Figure 11:**
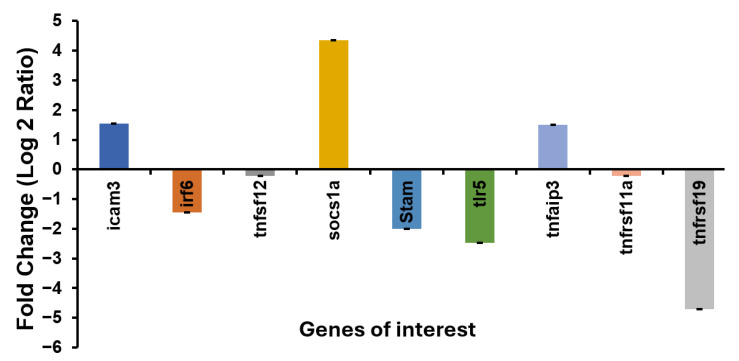
qRT-PCR validation of DEG expression profiles in ISKNV-infected ASB spleen. The qRT-PCT analysis was performed to validate the expression profiles of DEGs identified through RNA-seq in ISKNV-infected ASB spleen. Expression levels were normalized using 18S rRNA as an internal control. Statistical analysis is significant (*p* < 0.05).

To assess the reliability of the RNA-seq data, RT-qPCR was used to analyze the expression patterns of nine potent pathogenicity genes selected in the pathway analysis. The primer sets used for RT-qPCR were validated through a melt curve analysis to confirm the specificity of the amplification products (Figure S1). All genes showed similar expression patterns to those of the RNA-seq data (Figure 11).

## 4. Discussion

Asian seabass is a widely cultured species across Southeast Asia, and ISKNV is a significant pathogen in aquaculture, causing high mortality rates and severe economic losses in Asian seabass farming. Understanding the pathogenic mechanisms of the virus is crucial for identifying critical points in the infection process, which can provide insights into the development of effective prevention strategies and treatments. In our study, the DEGs identified through NGS provide a comprehensive overview of the transcriptional landscape of Asian seabass infected with ISKNV. A pathway analysis was further performed to understand the biological significance of these genes in ISKNV-infected Asian Seabass. Viral infection involves complex interactions between the host and the virus, affecting various cellular processes and signaling pathways that the virus exploits to enter, replicate, and evade the host immune system. In our study, we identified key genes from pathways associated with immune response, interferons (IFNs), Toll-like receptors (TLRs), endocytosis, cell communication, cell cycle arrest, and programmed cell death.

To identify key genes among the DEGs, we explored relevant pathways in the Reactome database for both humans and zebrafish to understand the biological processes involved. Subsequently, we constructed a protein–protein interaction (PPI) network for Asian seabass using the STRING database and performed clustering to identify groups of interacting proteins. To identify hub genes, we applied six different algorithms. However, it is important to note the limitation that hub genes within the PPI network often participate in a single process or complex, which can lead to functional redundancy. Their removal might not significantly impact the network due to compensatory interactions by other proteins. Additionally, PPI data can be biased toward well-studied proteins, leading to an overrepresentation of certain hubs. This bias may limit the discovery of novel interactions. To mitigate these limitations, we propose selecting hub genes from different functional clusters. Genes that are not hub genes but are connected to different functional clusters or pathways may still hold significant importance. In addition, the PPI network of Asian seabass was compared with those of zebrafish and human to identify missing and disconnected proteins in Asian seabass, integrating findings from related functional studies in the literature to provide a comprehensive analysis.

In our study, 69 key genes were identified, with 41 of them being hub genes in the PPI network. Hub genes often have numerous interactions with other genes, playing a central role in gene regulatory networks. The dysregulation of these hub genes in ISKNV-infected spleen cells may indicate their crucial roles in the infection process. In the endocytosis pathway, the hub gene AP2A1, a component of adaptor protein 2 (AP2), is involved in regulating viral entry. The knockdown of AP2-associated kinase 1 (AAK1) significantly decreased the infection of cells by Rabies virus (RABV) [9]. The interactions between alphaviruses nsP3 protein and SH3KBP1 are required for efficient infectivity [10], while another study showed that the degradation of SH3KBP1 can antagonize host innate immune responses [11]. In PI3K pathway, GAB1, an adaptor protein, facilitates PI3K activation and downstream signaling. The downregulation of GAB1 reduces PI3K/Akt activity, which may promote viral infection [12]. FGFR3, which regulates cell proliferation and survival via PI3K/Akt activation. The downregulation of FGFR3 may reduce host cell proliferation, allowing viruses to limit immune cell activation or promote immune evasion [13]. KL inhibits the PI3K/Akt/mTOR pathway and regulates cellular aging and immune responses. Its downregulation enhances PI3K/Akt signaling, promoting cell survival and viral replication [14]. PIK3R2, a key component of the PI3K pathway, suppresses PI3K/Akt activation when downregulated, potentially reducing viral replication [15]. Altogether, the hub genes demonstrating a role in viral infections will be prioritized for studying their effects on ISKNV pathogenesis.

In addition, 28 key genes were identified from missing, disconnected, or bridge genes linking different pathways or clusters. All these key genes have demonstrated their function in virus infection in various studies. The tumor necrosis factor receptor super family (TNFRSF) TNFRSF11A was not found in the Asian seabass PPI network, while it interacts with almost all the hub genes of the shared immune genes network in human and zebrafish. It is linked with the TLR pathway hub gene BIRC2 by interacting with TNFSF12. The expression of TNFSF12 by macrophages in response to infection helps to modulate the innate inflammatory response [16]. Therefore, both TNFRSF11A and TNFSF12 may influence ISKNV pathogenesis in Asian seabass. TNFRSF19 is associated with pro-apoptosis [17]. IRF6 was identified as a positive regulator of IFN expression and is involved in both MyD88 and TBK1 pathways in fish [18]. In our study, both genes were downregulated, potentially facilitating ISKNV infection. SYNJ1 can limit the uptake of *Staphylococcus aureus* by counteracting the accumulation of PI-4,5-P2 [19], suggesting that SYNJ1 activity can impact the efficiency of pathogen entry into host cells. As part of antivirus response, virus infection will benefit from the downregulation of the IFN pathway. In our study, the key genes in the IFN pathway, NLRX1 and DHX9, were downregulated. Studies have shown that helicase DHX9 is crucial for antiviral responses due to its role in recognizing viral RNA [20], and NLRX1 promotes immediate IRF1-directed antiviral responses [21]. Studies have shown that the replication of coronaviruses were reduced in PIK3R4 KO cells [22], and the knockdown of TLR5 enhances pseudorabies virus (PRV) replication [23]. In our study, TLR5 was downregulated, while PIK3R4 was upregulated, both demonstrating a pro-infection activity. ATP6V1A facilitates rabies virus replication [24], and its ortholog, ATP6V1AB, may have a similar effect on ISKNV pathogenesis. The gene AGTR1b, part of the actin nucleation cluster, may drive COVID-19 pathology [25]. In the cell communication pathway, PPIB is critical for the efficient replication of the hepatitis C virus (HCV) genome [26]. In our study, PPIB transcription was also upregulated, suggesting its potential role in ISKNV replication. Fibulin-1 (FBLN1) has demonstrated the ability to inhibit E6-mediated transformation in cells infected with human papillomavirus (HPV) [27], while F11r.1 (also as JAMA), part of the leukocyte cell–cell adhesion cluster, plays an important role in rotavirus entry into host cell [28].

**Figure 12:**
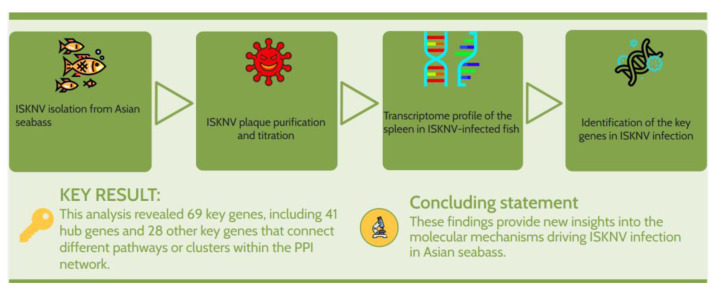
Schematic diagram summarizing the study workflow. This diagram outlines the key experimental steps, including ISKNV isolation from infected Asian seabass, viral plaque purification and titration, followed by transcriptome profiling of spleen tissue from experimentally infected Asian seabass. The study emphasizes the identification of key genes associated with ISKNV infection, providing insights into the molecular mechanisms of infection in Asian seabass.

Overall, the identified DEGs provide a comprehensive overview of the host transcriptomic response to ISKNV infection. The relationship between the viral genes carried by ISKNV and the host’s immune-related gene behavior is a fascinating area of study. Like other iridoviruses, ISKNV employs various strategies to evade or suppress the host’s immune response, ensuring its survival and replication within the host. Understanding these interactions is crucial for developing effective strategies to combat ISKNV infections. For instance, targeting viral immune repressor genes or reversing their effects on host immune pathways could pave the way for novel therapeutic approaches. Our findings provide valuable insights into the molecular mechanisms driving ISKNV infection in Asian seabass (Figure 12). One potential limitation of our study is the lack of biological replicates, as only one tank was used per experiment. While the environmental parameters (including water quality, humidity, temperature, light cycle, and feeding cycle) were precisely controlled, including multiple tanks per treatment would be essential for capturing variability across different tanks and ensuring that observed effects are consistently attributable to the virus challenge rather than tank-specific factors. Future research should focus on elucidating the precise regulatory functions of these key genes and exploring their effects on ISKNV infection in Asian seabass. Prior to this, all key genes will undergo rigorous validation through RT-qPCR, including amplification efficiency testing and the incorporation of additional biological replicates. These findings not only expand our understanding of ISKNV pathogenesis but also pave the way for the development of potential targeted therapeutic interventions to enhance disease resilience in aquaculture species.

## Figures and Tables

**Table 1:** Primers for DEG verification by RT-qPCR.

Primer	Oligonucleotide Sequence (5′–3′)
IRF6-F	TTGGTTAAGCAGGCCGAGAG
IRF6-R	ATCCACACCAGACCCGGATA
ICAM3-F	CTGCATCCGTGTCTCCAACT
ICAM3-R	TTGATACCACCGCACAACGA
TNFRSF19-F	GGTTATGGAGAGGATGCCCG
TNFRSF19-R	CCCTTCTGGAAGCGGTTGAT
TLR5-F	CAGGATGAAACAAATCCCCAGC
TLR5-R	TGTTCAGGTCTGTCTGGAGC
SOCS1A-F	GCCAAAAGTTCTCACTGGCG
SOCS1A-R	GTATGGGGTGCTGAGGCTTT
STAM-F	ATGACGCAGACGCCAAACTA
STAM-R	AGGACTGGTGCATCTGTGTG
TNFAIP3-F	CCTTTGCCCAGAGTGCCATA
TNFAIP3-R	TCTTGGTTGGCGTAGTGGTC
TNFRSF11A-F	AATACCAGCCCAGCTTGACC
TNFRSF11A-R	ATGGGACAGCATTGGAGGTG
TNFSF12-F	GCAGGCGTCTACTTCCTGTT
TNFSF12-R	CAAAGTGGAAAGAGTGCGGC
18S-F	AACGAGACTCCGGCATGCTA
18S-F	CCGGACATCTAAGGGCATCA

## Data Availability

Data will be made available upon request. The datasets presented in this study can be found in online repositories. The names of the repository/repositories and accession number can be found in the article.

## Supplementary Materials

The following supporting information can be downloaded at: https://www.mdpi.com/article/10.3390/v17050728/s1. Figure S1: Melt curve analysis of the RT-qPCR validation; Table S1: Primers used for identifying ISKNV in this study; Table S2: Homology analysis and gene list for segregation in REACTOME; Table S3: DEGs in the spleen of Asian seabass infected with ISKNV; Table S4: DEGs segregated according to the biological pathways using the Reactome server; Table S5: String interactions for the 31 shared immune genes; Table S6: String interactions of the genes in the IFN pathway; Table S7: String MCL clusters for the IFN signal; Table S8: String interactions of the genes in the TLR pathway; Table S9: String MCL clusters for TLR signal; Table S10: String interactions of the genes in endocytosis pathway; Table S11: String MCL clusters for the endocytosis signal; Table S12: String interactions for cell–cell communication; Table S13: String MCL clusters for cell–cell communication; Table S14: String interactions for PCD; Table S15: String MCL clusters for PCD; Table S16: String interactions for cell cycle arrest; Table S17: String MCL clusters for cell cycle arrest; Table S18: String interactions of the genes in the PI3K pathway; Table S19: String MCL clusters for PI3K signal.
