# Supplementary figures and images for: Transcriptomic Analysis of the Spleen from Asian Seabass (Lates calcarifer) Infected with Infectious Spleen and Kidney Necrosis Virus
Source: Viruses. 2025 May 19;17(5):728. doi: 10.3390/v17050728 (PMC12115965; doi:10.3390/v17050728)

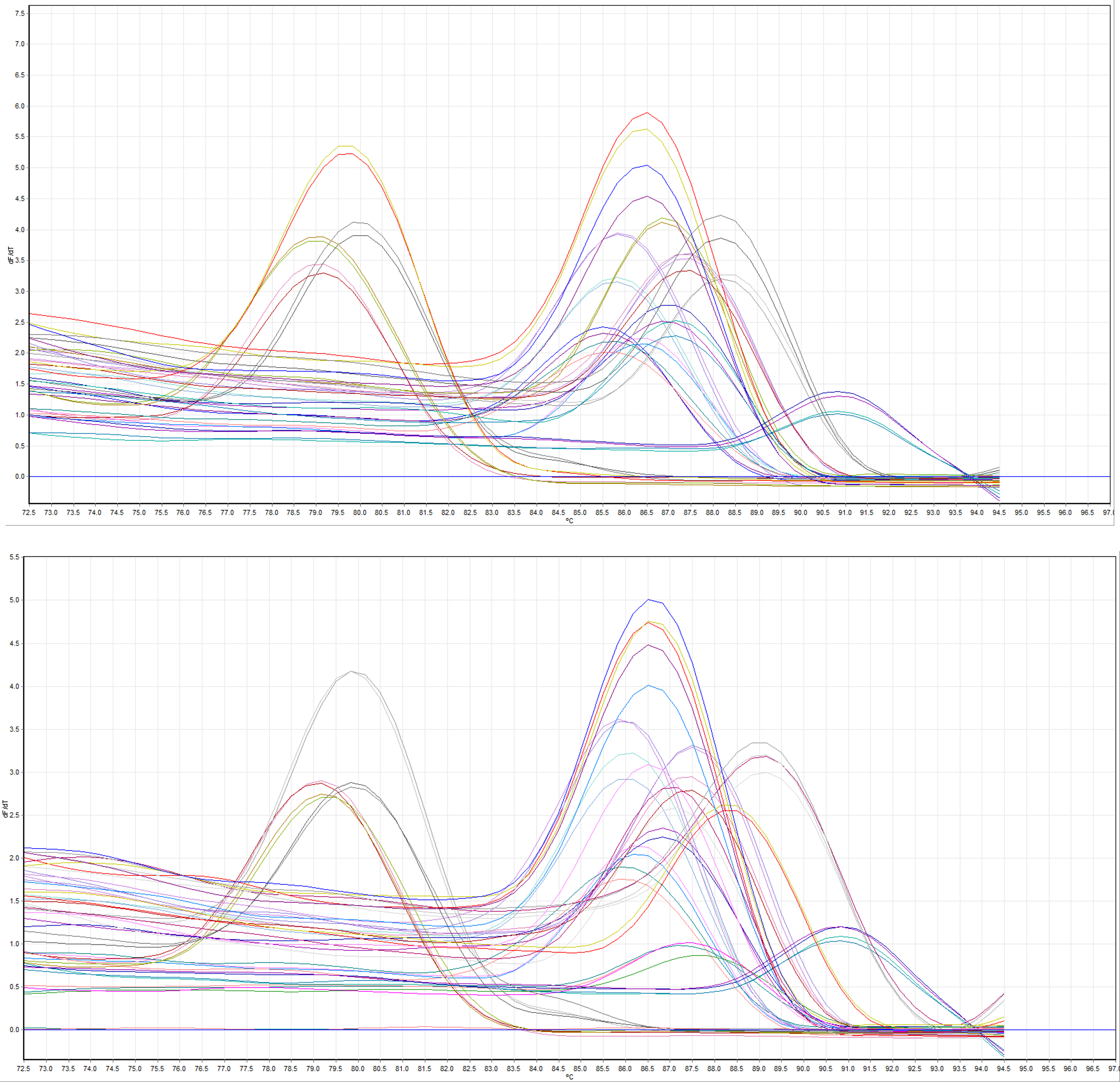

Supplement: Supplementary file 1 [file viruses-17-00728-s001.zip › Figure S1.png]
